# What is the potential for plural ownership to support a more inclusive economy? A systematic review protocol

**DOI:** 10.1186/s13643-022-01955-y

**Published:** 2022-04-23

**Authors:** Elaine Tod, Deborah Shipton, Gerry McCartney, Shifa Sarica, Graeme Scobie, Jane Parkinson, Anne-Marie Bagnall, Julian Manley, Andrew Cumbers, Sarah Deas, James de le Vingne

**Affiliations:** 1 Public Health Scotland, Glasgow, Scotland, UK; 2 University of Glasgow, Glasgow, Scotland, UK; 3 Leeds Beckett University, City Campus, Leeds, England, UK; 4 University of Central Lancashire, Preston, England, UK; 5 Wellbeing Economy Alliance, Edinburgh, Scotland, UK; 6 Employee Ownership Association, Brough, England, UK

## Abstract

**Background:**

The world is facing an unprecedented systemic shock to population health, the economy and society due to the devastating impact of the COVID-19 pandemic. As with most economic shocks, this is expected to disproportionately impact vulnerable groups in society such as those in poverty and those in precarious employment as well as marginalised groups such as women, the elderly, Black, Asian and Minority Ethnic (BAME) groups and those with health conditions. The current literature is rich in normative recommendations on plural ownership as a key building block of an inclusive economy rooted in communities and their needs. There is, however, a need for a rigorous synthesis of the available evidence on what impact (if any) plural ownership may potentially have on the inclusivity of the economy. This review seeks to synthesise and compare the available evidence across the three economic sectors (private, public and third).

**Methods:**

We will search eight bibliographic databases (Sociological abstracts, EBSCO Econlit, OVID Embase, OVID Medline, Applied Social Sciences Index and Abstracts (ASSIA), ProQuest Public Health, Web of Science, Research Papers in Economics (Repec) – EconPapers) from the earliest data available in each database until January 2021. Grey literature will be identified from Google (advanced), Google Scholar and 37 organisational websites identified as relevant to the research question. We will include comparative studies of plural ownership from high-income countries that report outcomes on access to opportunities, distribution of benefits, poverty, and discrimination. A bespoke search strategy will be used for each website to account for the heterogeneity in content and search capabilities and will be fully documented. A standardised data extraction template based on the Population-Intervention-Context-Outcome (PICO) template will be developed. We will assess the strength of evidence for different forms of economic ownership identified in relation to the impact of each on the four economic outcomes of interest, paying particular attention to the role of wider contextual factors as they emerge through the evidence.

**Discussion:**

The findings of this review are intended to inform policymaking at local, national and international level that prioritises and supports the development of different economic and business models.

**Systematic review registration:**

Open Science Framework registration DOI: 10.17605/OSF.IO/BYH5A

**Supplementary Information:**

The online version contains supplementary material available at 10.1186/s13643-022-01955-y.

## Background

The world is facing an unprecedented systemic shock to population health, the economy, and society due to the devastating impact of the COVID-19 pandemic. The magnitude of the impact on the economy globally and in Scotland is not yet fully understood but is likely to be far-reaching with a ripple-effect that will be felt for years to come. As well as the large national deficit, the country is likely to face a sharp recession. As with most economic shocks this is expected to disproportionately impact vulnerable groups in society such as those in poverty and precarious employment [1] as well as marginalised groups such as women, older people, BAME groups and those with health conditions.

Many will be aiming to “bounce back” to the economy pre-crisis as soon as possible. However, the economic and social challenges we face, as well as the continued threat of the resurgence of COVID-19 infections means that now more than ever we need to learn and adapt to be better able to manage the challenges ahead. There is much we can draw on from the work pre-crisis on designing a more resilient, inclusive economy for the future; one that embeds the economy back into society and protects those who are most vulnerable [2].

To achieve a Scotland where the population and the economy thrives, structural changes are needed. The Scottish National Performance Framework (SNPF) [3] already sets out an overall purpose and vision for Scotland; a key part of which is to create opportunities for Scotland to flourish through increased wellbeing and sustainable and inclusive economic growth. The delivery of a sustainable and inclusive economy is also one of the six national public health priorities [4], reflecting the fundamental role that inequalities in income, wealth and power have on causing poor health and health inequalities.

Plural ownership has been identified as a key building block towards a more inclusive economy, potentially delivering a more equitable distribution of income, wealth and power. It refers to a broadening of the models of ownership of the production and sales of goods and services to distribute ownership and control among a greater range of stakeholders, such as employees, customers, producers or the community. It has been described as a:

“broadening of the different types of enterprises that serve our local economies, so that there are more community-embedded forms of business ownership which are more likely to generate wealth for local residents and communities.” (Leibowitz, CLES 2019) [5]

Evidence suggests that plural ownership can deliver a number of benefits. These include wage growth, job growth and security, increased employee control, increased accessibility to the job market for marginalised populations, community cohesion and resilience, increased business survival, and retained wealth within the community. Reflecting the potential for plural ownership of the economy to deliver such benefits, it is included as one of the five pillars of community wealth building [6].

**Fig. 1:**
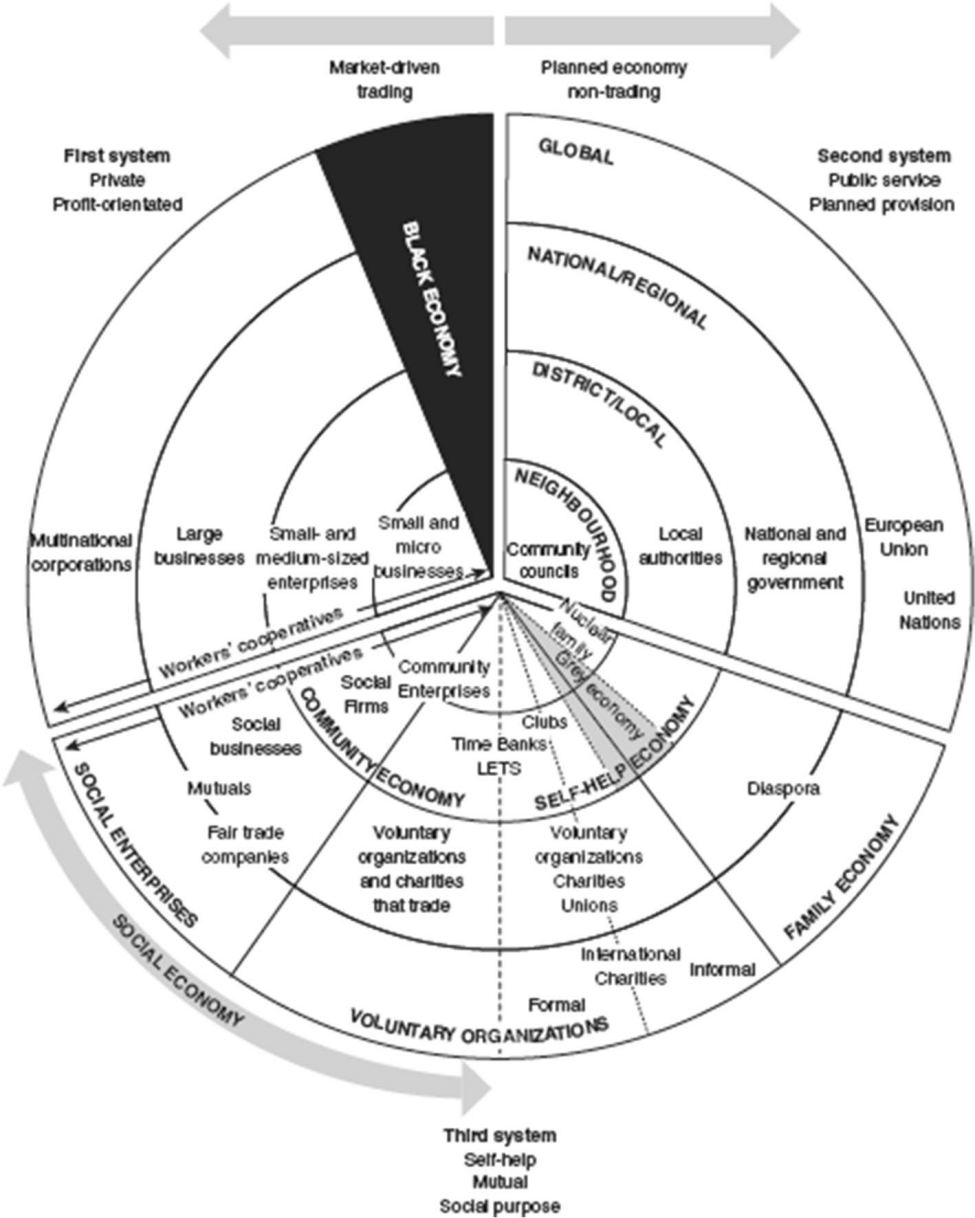
Pearce’s model of the economy [7]. Source: Pearce, J. Social Enterprise in Anytown. 2003 (p25) *Reproduced with written permission from the Calouste Gulbenkian Foundation, London*

One way of representing the economy is using Pearce’s model (Fig. 1). This illustrates the breadth of ownership models found in each of the three sectors (systems) and will be used as a framework for organising the main ownership models identified for each sector as they emerge. It is not intended to be exhaustive as reviewing the evidence in detail for every model is beyond the scope of this review.

The current literature is rich in normative recommendations for plural ownership as a means of fostering a more inclusive economy through a focus on communities and their needs. There is however a need for a rigorous synthesis of the available evidence on what impact (if any) plural ownership may potentially have on community wealth and the drive towards a more inclusive economy. This review seeks to synthesise the available evidence across the three sectors (private, public and third) and what is known about the impact of different forms of ownership in each sector on the development of a more inclusive economy.

### Objectives

The research question was formed using a combination of the SPICE (setting, population/problem, intervention, comparison, evaluation) and PICO (population, intervention, context and outcome) approaches.

The following research question will form the basis for this systematic evidence review: What is the evidence from comparative studies that plural forms of economic ownership, without restrictions in time, person or place, have differential impacts on a broad range of inclusive economic outcomes, and thereby might support change towards a more inclusive economy?

## Methods

We developed this protocol in accordance with PRISMA-P 2015. See Additional file 1 for the PRISMA-P checklist.

### Eligibility criteria

The inclusion and exclusion criteria for screening of studies were informed by the PICOS framework and are shown in Table 1.

**Table 1:** Inclusion and exclusion criteria

	Inclusion criteria	Exclusion criteria
**Population**	Communities in high-income countries (as per World Bank world “Atlas method” definition^a^, June 2020)	Low- and middle-income countries
**Intervention**	Ownership models under the three systems (private, public and third) as defined by Pearce	None
**Comparator**	1. A different ownership model to intervention in study 2. A before and after comparison 3. Different population counterfactual	No comparator
**Outcomes**	Access to opportunities Distribution of benefits Poverty Discrimination	Outcomes not included in the Inclusive Economy definition (see Appendix)
**Study type**	Quantitative, systematic review, meta-analysis, descriptive (with comparator), observational studies, experimental studies, natural experiments	Commentaries, editorials, descriptive studies without any comparator, studies that do not draw on empirical data, qualitative studies
**Language**	English only	Studies not written in the English language
**Time-frame**	Earliest available to January 2021	None
**Publication status**	Both published, unpublished and on-going studies	None

^a^High-income economies are those in which 2019 GNI per capita was $12,536 or more. https://datahelpdesk.worldbank.org/knowledgebase/articles/906519

### Information sources

According to Lampel [8] (in Whittam and Talbot [9]), the nature of employee ownership (and by extension wider forms of ownership) means that the topic draws a wide range of interest across multiple disciplines and bodies, as such identifying key sources of evidence may be challenging. Balancing this with the need to be mindful of resource constraints, we will search the following sources:

Sociological abstracts: EBSCO Econlit; OVID Embase; OVID MEDLINE; Applied Social Sciences Index and Abstracts (ASSIA); ProQuest Public Health; Web of Science; Research Papers in Economics (Repec)–EconPapers; Fraser of Allander Institute; Joseph Rowntree Foundation; Centre for Local Economic Strategies (CLES); Scottish Government; Democracy Collaborative; NCEO-National Center for Employee Ownership; The Next System Project; Power to Change; United Nations (UN); Organisation for Economic Cooperation and Development (OECD); OECD library; World Bank; International Monetary Fund (IMF); International Labor Organization; Trade Unions Congress (TUC); European Commission; New Economics Foundation; Business in the Community; Carnegie UK Trust; Economic and Social Research Council (ESRC); Employee Ownership Association; Co-operatives UK; London School of Economics; Nesta; Forum for the Future; World Health Organization (WHO); Esmée Fairbairn Foundation; Centre for Thriving Places; Scottish Community Development Centre; Social Enterprise Scotland; Social Enterprise UK; Social Finance; World Cooperative Monitor; UWS-Oxfam Partnership; International Finance Corporation; Business for Inclusive Growth; WISE Campaign.

### Search strategy

We built up the key search terms for the bibliographic database searches using the PICO framework (Table 2). Included terms were informed by literature identified from the initial screening pilot, the pilot search strategy tested across all eight databases, feedback from the expert advisory group and keywords assigned to relevant papers. The terms were then coded as necessary to allow each database to be searched.

**Table 2:** Concept table of search terms (PICO) for peer-reviewed search

(P)opulation	(I)ntervention	(C)omparator	(O)utcome
High-income countries (defined according to World Bank “atlas method” classification) *Papers will be filtered by location during the screening phase	**Plural ownership** “plural ownership” or “equitable business ownership” or “distributed ownership or “distributed control” or democratic control” **First system—private/profit oriented** “Multi-national corporation” or “large business” or “small and medium sized enterprise” or “small and micro business” Or B corporation” **Second system—public services/planned provision** “Public sector” or “national government” or “regional government” or “local authority” or “community council” or municipal or “state ownership” or “anchor institution” **Third system—self-help/mutual/social purpose** “third sector” or “third system” or “nongovernmental organization*” or “nongovernmental organisation*” or “nonprofit organization*” or “nonprofit organization*” or “voluntary sector” or “civic sector” or “nonprofit sector” or “community sector” or “public enterprise*” or “public-private enterprise*” or “private enterprise*” or “formal sector” or “informal sector” or “shadow economy” or “informal institution* or cooperative or co-operative or “worker cooperative” or “worker co-operative” or “housing cooperative” or “housing co-operative” or “employee ownership” or “consumer retail cooperative” or consumer retail co-operative” or “neighbourhood cooperative” or “neighbourhood co-operative” or “credit union” or “common ownership company” or community development finance institution” or “social business” or “social firm” or “social enterprise” or “community enterprise” or voluntary enterprise” or “charity” or “club” or “time bank” or “local exchange trading scheme” or “family business”)	*1. A different ownership model to intervention in study* *2. A before and after comparison* *3. Different region counterfactual*	**Inclusive economy** economic democracy or economic equality or solidarity economy or fair economy or just economy or economic inclusion or inclusive economy or inclusive growth or pro-poor growth or shared prosperity or shared growth or equity or justice or equality or inequality or economic inequality or economic resilience or wellbeing economy or well-being economy or resilient cities or urban resilience or resilient development or inclusive institution or economic impact or impact on economy **1) Equal access to opportunities:** “access to opportunities” or “power inequality” or “increase* participation in the economy” or “economic participation” or “good work” or “fair work” or “fair income” or “job security” or “access to education” or “access to training” or “investment” or “innovation” **2) Equitable distribution of the benefits of the economy** “fair distribution of benefits” or “income growth” or “income inequality” or “wealth inequality” or “distribution of wealth” or “distribution of income” or “affordable cost of living” or “basic needs” or “local wealth retention” “asset equality” or “asset inequality” or asset distribution” or “financial inclusion” or “access to finance” or “access to capital” **3) Reduced poverty** poverty or “poverty reduction” or reduc poverty or “basic needs” or “sustainable” **4) Reduced discrimination** reduc* discrimination or reduc* oppression or “social justice”

The key terms used to search for grey literature using Google and Google Scholar are shown in Table 3. Due to the limitations in terms of the use of Boolean operators and word limits in a single search using Google, it was necessary to condense the search to a more manageable list. This list focused on higher level terms for each of the three economic systems the review is focussing on and key ownership models. Each of these models (Search A) was combined separately with Searches 1–4 shown on the right hand side of Table 3 to limit the searches to results which focused on both the intervention and outcome.

**Table 3:** Grey literature search: high-level search terms for Google Scholar and Google

Plural ownership and sectors (intervention)		Inclusive economy (outcome)
**Search A** (“plural ownership” | “democratic ownership” | “equitable ownership” | “private sector” | “private ownership” | “private enterprise” | “b-corporation” | “public sector” | “state ownership” | “third sector” | co-operative | “employee ownership” | “social enterprise”)	**AND**	**Search 1** (“inclusive economy” | “inclusive growth” | “wellbeing economy”) **Search 2** (“fair economy” | “community wealth building” | inequality | poverty) **Search 3** (“wealth retention” | discrimination | “distribution of benefits”) **Search 4** (“access to opportunities” | “local jobs” | “fair wage”)

*Search term combinations*: Search A + Search 1; Search A + Search 2; Search A + Search 3; Search A + Search 4. Searches were restricted to the English language, PDF format

When searching each website for grey literature a flexible approach using multiple search pathways was used due to the heterogeneous nature of website content, design and variations in search functionality. This ensured that each website was interrogated effectively. The search pathways used for each website will be clearly documented as part of the systematic review.

### Study records

#### Data management

The Refworks [10] reference management tool will be used for importing, storing and de-duplicating peer-reviewed literature from bibliographic databases. The Sciwheel [11] database will be used for storing and de-duplicating grey literature and extracting details of studies from websites. The Covidence [12] systematic review management tool will be used to manage title/abstract screening, full text review, and data extraction stages of all references (peer-reviewed and grey literature) in this systematic review.

#### Selection process

All peer-reviewed studies will go through a blind dual-screening process of both the title and abstract and then full-text of all remaining eligible studies. Disagreements will be resolved by consensus, and if necessary arbitration of a third person. Papers excluded at the full-text screening stage will be detailed in an Appendix with the reason for the exclusion. Grey literature will be selected in two ways:

- Google and Google Scholar
  The first 100 PDF results returned for each search on Google and Google Scholar (excluding minutes and book reviews) will be imported to Sciwheel and will go through blind dual title and abstract screening.
- Websites
  Due to the heterogeneous nature of the websites potentially relevant to this review and inherent difficulties in dual-screening, we will single-screen websites for relevant content from title/abstract or summary equivalent, documenting all approaches used to interrogate each website separately and recording each search for transparency. Grey literature selected in this way at the ‘title/abstract’ stage will then be imported to Covidence systematic review management software to the full-text screening stage. All imported grey literature will then be dual-screened at the full-text screening stage.

### Data collection process

A standardised data extraction template will be piloted and refined in Covidence. For the pilot, ten studies will be chosen randomly for data extraction by two reviewers. Both reviewers of each paper will independently extract key information from each paper deemed eligible from the full-text screening stage. Where any key information is missing from a paper, we will attempt to contact the author(s) for clarification.

### Data items

The key data points to be extracted from each paper are shown below.

- Administrative data (author, institution(s), date of study, funding source)
- Study characteristics
  - ° Type of study (drop down list)
  - ° Population/setting for study
  - ° Intervention(s)
  - ° Comparator(s)
  - ° Outcome(s)
  - ° Is study reported more than once?
    - ▪ If yes, which study was retained for the review and why?
- Methods
- Key results
- Key conclusions
- Quality of underlying evidence (risk of bias, confounding, gaps in evidence)
- Contextual factors (e.g. the balance between economic systems, regulation, financial support, labour etc.)

## Outcomes and prioritisation

This review will focus on four key economic outcomes from our working inclusive economy definition necessary (though not sufficient) to help support a more inclusive economy (see Appendix for full definition). Any data on these four outcomes will be recorded with no restriction on the direction of impact. They are all main outcomes and will be treated with equal priority.

1. Access to opportunities
  These include:
  i. Education, training, employment
  ii. Owning and running businesses
  iii. Owning and managing community assets
  iv. Finance (to facilitate the above)
  Access to opportunities will also depend on removal/lack of barriers to opportunities, such as social or cultural barriers.
2. Distribution of the benefits of the economy
  i. Resources, such as good quality jobs, wealth, assets and living standards
  ii. Distribution of power including: economic power, power related to the generation and communication of knowledge, including the media, power around shaping cultural and beliefs systems, power around shaping institutional cultures (related to item 1) collective power and positional power.
  iii. The presence or absence of exploitative practices in relation to plural ownership.
3. Poverty
  i. Data on levels of poverty in society.
4. Discrimination
  i. Impact of plural ownership (if any) on both active and passive discriminatory practices.

## Risk of bias in individual studies

From the initial scoping exercise, it is expected that the majority of eligible studies returned will be from non-randomised studies, observational studies or reviews. A modified version of the Effective Public Health Practice Project (EPHPP) tool [13] will be used for assessing the risk of bias in individual outcomes from each study. EPHPP is a well-recognised, validated tool for assessing risk of bias in quantitative studies and covers six domains (selection bias, study design, confounders, blinding, data collection methods and withdrawals and dropouts). The main modification to this tool will be to allow for different units of observation with studies at an ecological level more likely than those at an individual level.

For systematic reviews, we will use ‘A MeaSurement Tool to Assess systematic Reviews 2’ (AMSTAR2) [14] criteria which was developed for systematic reviews that include non-randomised studies. Risk of bias assessment will be incorporated into the data synthesis through narrative discussion.

## Data synthesis

We will assess the strength of evidence for different forms of economic ownership identified from the literature in relation to the impact of each on the four economic outcomes of interest, paying particular attention to the role of wider contextual factors as they emerge through the evidence, e.g. the balance between economic systems, regulation, financial support, and labour. We will aim to distinguish between the evidence of impact for different discrete models where there are data available but this will be non-systematic.

Due to the anticipated heterogeneity of interventions, outcomes, comparators and contextual factors across studies, it will not be feasible to undertake a meta-analysis of results. Instead, we will carry out a narrative synthesis of the evidence and will report this in accordance with the Synthesis WIthout Meta-analysis (SWiM) guidelines. Pearce’s three economic systems model will be used as an economic framework (private, public and third systems) to help guide the synthesis.

## Confidence in cumulative evidence

Formal assessment of the quality of the evidence will be undertaken using the *Grading of Recommendations Assessment, Development and Evaluation* (GRADE [15]) approach where possible and appropriate. GRADE looks at the overall quality of evidence relating to each outcome of interest as opposed to each study. The quality of evidence for each outcome may vary. This will be used in the synthesis of information in this review.

## Dissemination of findings

We will disseminate our findings through peer-reviewed publication as well as an accessible publication, through discussions with interested partners, and via our expert advisory group.

## Target audience

North Ayrshire Council, other local authorities, Scottish Government, national and international policy makers and practitioners, academics, third sector bodies.

## Governance

The development of the systematic review protocol and the systematic review will be guided by the internal project management team (led by Public Health Scotland) and the expert advisory group.

## Pilot study

### Initial scoping of topic area

An initial scoping exercise (review of key papers identified a priori, search of Google and key organisational websites) was carried out to identify studies which had sought to review or generate evidence on different forms of economic ownership and their inclusive economic impact. From this, we identified gaps in evidence which we used to formulate the research question for our proposed systematic review.

### Pilot search strategy

We carried out a pilot search for peer-reviewed literature using search terms collated from literature identified from the scoping search and feedback from the expert advisory group.

Both sensitive and specific versions of the searches were then run on all eight databases identified as potentially relevant to the research question and key search terms were further refined i.e. ambiguous terms were either removed or revised to keep the study focussed on the research question. The sensitivity of the search was tested by looking for the inclusion of key papers (identified from the initial scoping study) in the results. The search strategy used for the EBSCO Econlit database, as an example, is shown in Additional file 2.

### Supplementary Information

**Additional file 1.** PRISMA-P-checklist.

**Additional file 2.** Econlit (EBSCOhost)s–Search strategy.

## Data Availability

Not applicable.

## Appendix

### Defining an inclusive economy

As with emerging concepts, there is no one uncontested definition of what an inclusive economy is, how it should be achieved or what difference we would see in the economy if we were to achieve a more inclusive economy. The theory of how the current system has failed to deliver equity and economic performance is still emerging, which is possibly why there is no clear definition of an inclusive economy.

For this review, we need a working definition of an “inclusive and sustainable economy”. Our working definition doesn’t make the claim that the included concepts are necessary or sufficient for an inclusive and sustainable economy. The emerging evidence base and the work that we carry out going forward will be used to define our definition.

Drawing on a synthesis of the literature the NHS PHS (National Health Service Public Health Scotland) working definition of an “inclusive and sustainable economy” is one in which:

1. The institutions, governance and goals of the economy are designed to deliver fairness, transparency and accountability.
2. There is equal access to opportunities; this includes access to:
  i. Education, training, employment owning and running businesses
  ii. Owning and managing community assets
  iii. Finance (to facilitate the above)
  Equal access to opportunities will also result from removal/lack of barriers to opportunities, such as social or cultural barriers.
3. There is equitable distribution of the benefits of the economy, identified as:
  i. Resources, such as good quality jobs, wealth, assets and living standards
  ii. Equitable distribution of power including: economic power, power related to the generation and communication of knowledge, including the media, power around shaping cultural and beliefs systems, power around shaping institutional cultures (related to item 1) collective power and positional power.
  iii. Equitable distribution will also require an absence of exploitative practices.
4. There is reduced levels of poverty in society,
5. There is an absence of both active and passive discriminatory practices,
6. Operates within planetary boundaries.
7. Reduce negative impact of intersectionality
