# Supplementary material for: What is the potential for plural ownership to support a more inclusive economy? A systematic review protocol
Source: Syst Rev. 2022 Apr 23;11:76. doi: 10.1186/s13643-022-01955-y (PMC9034259; doi:10.1186/s13643-022-01955-y)
Supplement: Supplementary file 2 — Additional file 2. Econlit (EBSCOhost)s–Search strategy. [file 13643_2022_1955_MOESM2_ESM.docx]

Econlit (EBSCOhost) – Search strategy

Search date: 21 September 2020

Search period: 1945-2020

| **#** | **Search terms** | **Search type** | **Results** |
| --- | --- | --- | --- |
| S8 | S5 AND S6 | **Narrow by Language:**- english  **Search modes** - Boolean/Phrase | 2,732 |
| S7 | S5 AND S6 | **Search modes** - Boolean/Phrase | 2,727 |
| S6 | S1 OR S2 OR S3 OR S4 | **Search modes** - Boolean/Phrase | 41,838 |
| S5 | TI( “economic democracy” or “economic equality” or “solidarity economy” or “fair economy” or “just economy” or “economic inclusion” or “inclusive econom*” or “inclusive growth” or “pro-poor growth” or “shared prosperity” or “shared growth” or equity or justice or equality or inequalit* or poverty or “poverty reduction or “reduc* poverty” or “economic inequalit*” or “income inequalit*” or “power inequalit*” or “wealth inequalit*” or “income equalit*” or “power equalit*” or “wealth equalit*” or “distribution of power” or “distribution of wealth” or “distribution of income” or “resilient econom*” or “economic resilience” or “wellbeing econom*” or “well-being econom*” or “resilient cit*” or “urban resilience” or “resilient development” or “inclusive institution*” or “economic impact” or “impact of the econom*” or “increas* access to opportunities” or “increas* participation in the economy” or “economic participation” or “equitable distribution of benefits” or “social justice” or “good work” or “fair work” or “fair income” or “job security” or “financial inclusion” or “reduc* discrimination” or “reduc* vulnerability” or “address* intersectionality” or “reduc* intersectionality” or “reduc* oppression” or “reduc* exploitat*” or “access to education” or “access to training” or “plural ownership” or “distributed ownership” or “distributed control” or “democratic control” or “asset equality” or “asset inequalit*” or “distribution of asset*” or “fair distribution of benefits” or “income growth” or “equitable ownership” or “democratic ownership” or investment or innovation or “labor share of income” or “labour share of income” or “labor share of GDP” or “labour share of GDP” or “affordable cost of living” or “local wealth retention” or “access to finance” or “access to capital” or “basic needs” or sustainable) OR AB( “economic democracy” or “economic equality” or “solidarity economy” or “fair economy” or “just economy” or “economic inclusion” or “inclusive econom*” or “inclusive growth” or “pro-poor growth” or “shared prosperity” or “shared growth” or equity or justice or equality or inequalit* or poverty or “poverty reduction or “reduc* poverty” or “economic inequalit*” or “income inequalit*” or “power inequalit*” or “wealth inequalit*” or “income equalit*” or “power equalit*” or “wealth equalit*” or “distribution of power” or “distribution of wealth” or “distribution of income” or “resilient econom*” or “economic resilience” or “wellbeing econom*” or “well-being econom*” or “resilient cit*” or “urban resilience” or “resilient development” or “inclusive institution*” or “economic impact” or “impact of the econom*” or “increas* access to opportunities” or “increas* participation in the economy” or “economic participation” or “equitable distribution of benefits” or “social justice” or “good work” or “fair work” or “fair income” or “job security” or “financial inclusion” or “reduc* discrimination” or “reduc* vulnerability” or “address* intersectionality” or “reduc* intersectionality” or “reduc* oppression” or “reduc* exploitat*” or “access to education” or “access to training” or “plural ownership” or “distributed ownership” or “distributed control” or “democratic control” or “asset equality” or “asset inequalit*” or “distribution of asset*” or “fair distribution of benefits” or “income growth” or “equitable ownership” or “democratic ownership” or investment or innovation or “labor share of income” or “labour share of income” or “labor share of GDP” or “labour share of GDP” or “affordable cost of living” or “local wealth retention” or “access to finance” or “access to capital” or “basic needs” or sustainable) | **Search modes** - Boolean/Phrase | 115,860 |
| S4 | TI(“third sector” or “third system” or “nongovernmental organization*” or “non-governmental organization*” or “nongovernmental organisation*” or “non-governmental organisation*” or “nonprofit organization*” or “non-profit organization*” or “nonprofit organisation*” or “non-profit organisation*” or “voluntary sector” or “civic sector” or “nonprofit sector” or “non-profit sector” or “community sector” or “public enterprise*” or “public-private enterprise*” or “private enterprise*” or “formal sector” or “informal sector” or “shadow economy” or “informal institution* or cooperative or “worker cooperative” or “worker co-operative” or “housing cooperative” or “housing co-operative” or “employee ownership” or “consumer retail cooperative” or “consumer retail co-operative” or “neighbourhood cooperative” or “neighbourhood co-operative” or “credit union” or “common ownership company” or “community development finance institution” or mutual or “social business” or “social firm” or “social enterprise” or “community enterprise” or “voluntary enterprise” or charity or club or “time bank” or “local exchange trading scheme” or “family business” or diaspora) OR AB(“third sector” or “third system” or “nongovernmental organization*” or “non-governmental organization*” or “nongovernmental organisation*” or “non-governmental organisation*” or “nonprofit organization*” or “non-profit organization*” or “nonprofit organisation*” or “non-profit organisation*” or “voluntary sector” or “civic sector” or “nonprofit sector” or “non-profit sector” or “community sector” or “public enterprise*” or “public-private enterprise*” or “private enterprise*” or “formal sector” or “informal sector” or “shadow economy” or “informal institution* or cooperative or “worker cooperative” or “worker co-operative” or “housing cooperative” or “housing co-operative” or “employee ownership” or “consumer retail cooperative” or “consumer retail co-operative” or “neighbourhood cooperative” or “neighbourhood co-operative” or “credit union” or “common ownership company” or “community development finance institution” or “social business” or “social firm” or “social enterprise” or “community enterprise” or “voluntary enterprise” or charity or club or “time bank” or “local exchange trading scheme” or “family business”) | **Search modes** - Boolean/Phrase | 9,734 |
| S3 | TI ( “public sector” or “national government” or “regional government” or “local authority” or “community council” or municipal* or “state owne*” ) OR AB ( “public sector” or “national government” or “regional government” or “local authority” or “community council” or municipal* or “state owne*” ) | **Search modes** - Boolean/Phrase | 23,656 |
| S2 | TI(“multi-national corporation” or “multinational corporation” or “large business” or “small and medium sized enterprise” or “small- and medium-sized enterprise” or “small and micro business” or “small- and micro-business” or “B corporation” or “private sector”) OR AB(“multi-national corporation” or “multinational corporation” or “large business” or “small and medium sized enterprise” or “small- and medium-sized enterprise” or “small and micro business” or “small- and micro-business” or “B corporation” or “private sector”) | **Search modes** - Boolean/Phrase | 10,869 |
| S1 | TI ( “plural ownership” or “equitable ownership” or “distributed ownership or “distributed control” or “democratic control” or “democratic ownership” ) OR AB ( “plural ownership” or “equitable ownership” or “distributed ownership or “distributed control” or “democratic control” or “democratic ownership” ) | **Search modes** - Boolean/Phrase | 28 |
